# Electrodeposition of Alloy Nanostructures (Co-Ni) in the Presence of Sodium Benzene Sulfonate (SBS) and Their Application in Alkaline Hydrogen Evolution

**DOI:** 10.3390/molecules30081771

**Published:** 2025-04-15

**Authors:** Aleksandra J. Domańska, Piotr M. Skitał

**Affiliations:** 1 Doctoral School of the Rzeszow University of Technology, 35-959 Rzeszów, Poland; 2 Faculty of Chemistry, Rzeszow University of Technology, 35-959 Rzeszów, Poland

**Keywords:** hydrogen evolution reaction, cobalt–nickel alloy, coating nanostructure, hydrogen overpotential, electrolysis efficiency

## Abstract

The galvanostatic electrodeposition of cobalt–nickel alloy coatings performed out on a 304 stainless steel substrate. The electrolyte baths contained metals salts, along with boric acid and sodium benzene sulfonate (SBS) as an organic additive in the deposition process. Structural and topographic analyses were performed using SEM-EDS and AFM techniques, respectively. The findings confirm the formation of nanostructured coatings. The images depicting various stages of coating formation indicated the inhibitory role of the organic additive. The presence of SBS enabled the formation of a coating composed of grains with diverse geometries and significantly reduced surface roughness. Hydrogen evolution was conducted in an alkaline environment (1 M NaOH). Overpotentials for the different structures were recorded at 10 mA/cm^2^, yielding 196 mV and 225 mV for the coatings deposited with and without SBS, respectively. Additionally, experiments were performed in a laboratory-designed electrolyzer, which allowed for the measurement of gas volumes (H_2_ and O_2_) generated under constant voltage and current conditions. The results demonstrated that the obtained coatings perform more effectively as hydrogen evolution cathodes than currently used materials, particularly under higher current densities. Electrolysis was conducted for 8 h, revealing improved stability of the coating deposited in the presence of SBS.

## 1. Introduction

Declarations and decisions regarding the energy transition of the European Union and other regions pose numerous challenges for scientists [1]. A significant portion of these challenges concerns hydrogen energy production [2,3]. Hydrogen has enormous potential as an energy carrier due to its high energy density of approximately 140 MJ/kg [4,5]. However, the transportation and storage of this gas remain key issues requiring the development of effective solutions [6]. The greatest expectations are associated with green hydrogen, which is produced through electrolysis powered by renewable energy sources (RES) [7,8]. This ensures environmentally friendly, zero-emission production of pure H_2_. Current research on electrolysis focuses on improving energy efficiency and reducing production costs [9]. Commonly used electrolyzers employ noble metals such as Pt [10] or Ir and Rh [11] as electrodes to enhance hydrogen evolution reaction (HER) efficiency. Unfortunately, these materials are scarce and significantly increase production costs [12,13,14]. It is important to note that process efficiency is influenced not only by the catalytic properties of the electrodes but also by the entire system. Electrolyzers inherently exhibit resistance, which lowers overall efficiency. As reported in a review paper [15], the total energy demand (ΔH) for hydrogen production ranges from 283.5 to 291.6 kJ/mol H_2_ in the temperature range of 0 to 1000 °C. Other studies [16,17] indicate that under standard conditions (25 °C), the energy requirement for producing 1 mole of H_2_ is 285.8 kJ, equivalent to 0.07939 kWh/mol H_2_. When converted to mass, this corresponds to 39.38 kWh/kg H_2_ or 25.39 g H_2_/kWh. It is important to recognize that these values assume 100% efficiency, while current technologies consume between 70.1 and 53.4 kWh to produce 1 kg of hydrogen [18], translating to an efficiency range of 56–74%.

Numerous studies on hydrogen evolution highlight the high catalytic activity of transition metals [19], such as cobalt [20,21] and nickel [22,23,24], used both individually and in alloyed forms [25,26,27,28]. Researchers have attributed the satisfactory performance of bimetallic transition metal alloys to their high conductivity and a large number of active sites [29]. In particular, the Co-Ni alloy exhibits enhanced efficiency due to lattice strain differences. Zhang et al. [30] have reported the high activity and stability of Ni-Co electrocatalysts not only in hydrogen evolution but also in the more demanding oxygen evolution reaction (OER). The catalytic activity for both HER and OER can be further improved through surface modifications [31]. Given the crucial role of cathode surface properties in hydrogen evolution, conductive substrates can be used for the deposition of metallic coatings [32]. The electrode surface should be sufficiently developed to increase the number of active electrochemical reaction sites [33]. At the same time, it should be noted that smoother surfaces facilitate faster and more efficient release of hydrogen gas. This is because smoother surfaces reduce diffusion resistance, allowing more efficient hydrogen release [34]. Rough surfaces, on the other hand, can impede release due to increased diffusion resistance and surface interactions [35].

Metallic coatings can be obtained through electrodeposition in solution, a technique characterized by its simplicity [36] despite the complexity of the process itself [37]. Multiple parameters influence the electrodeposition process, directly affecting the properties of the resulting coating [38,39]. Electroplating techniques often involve the introduction of additives to control deposition. The presence of additives in electrolytes promotes the formation of smooth, often glossy coatings [40]. As noted by Lv [41], coatings may contain inclusions from these additives, potentially affecting their properties. The effectiveness of an additive depends on its adsorption onto the cathode surface during electrodeposition [42]. Adsorbed additive molecules can alter activation energy and charge transfer kinetics while blocking active sites for adatom formation, thereby influencing the electrocrystallization mechanism and reducing nucleation rates [43]. A widely used additive is boric acid, which acts as a buffer in the cathode region [44,45,46]. Previous studies [47,48] have employed sodium benzene sulfonate (C_6_H_5_NaO_3_S) as an organic additive. The aromatic ring of this compound enhances mass transport from the electrolyte toward the electrode, thereby modifying electrodeposition kinetics. Meanwhile, lone electron pairs from oxygen atoms adsorb onto the cathode surface, blocking it [49]. This leads to a controlled deposition process and the formation of well-ordered crystalline networks. As a result, fine-grained coatings with improved mechanical properties can be obtained [50].

This study focuses on the electrodeposition of Co-Ni alloy coatings and the impact of SBS on their surface and catalytic properties. The coatings were evaluated as hydrogen evolution cathodes in an alkaline environment. The results were compared with platinum and stainless steel samples. The research presented in this work aims to contribute to increasing the share of electrolytic hydrogen in global production.

## 2. Results and Discussion

### 2.1. Coating Structures and Compositions

**Figure 1:**
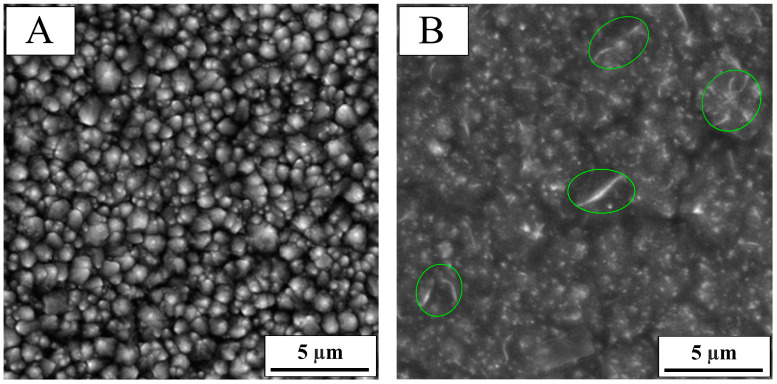
SEM images of alloy coatings: (**A**) Co-Ni, (**B**) Co-Ni(SBS), with a magnification of 10.0 kx. Characteristic elongated structures are highlighted with green circles.

SEM images of cobalt–nickel structures are presented in Figure 1, where image A shows the coating deposited without the additive, while image B represents the structure obtained in the presence of SBS. The applied current parameters and chloride-based electrolyte enabled the deposition of alloy coatings that achieved complete coverage of the chromonickel substrate. The coating consists of grains of varying diameters but with a uniform spherical shape. Comparing the SEM images, a distinct difference in the grain size distribution and morphology can be observed. Deposition in the presence of the organic additive allowed the simultaneous formation of spherical grains but also the formation of characteristic structures composed of elongated, sharply pointed grains, which are marked in Figure 1B. The coating deposited with the additive exhibited a glossy appearance, as indicated by the brighter, spherical structures. This confirms the smoothing effect of SBS, which slows down grain growth.

**Figure 2:**
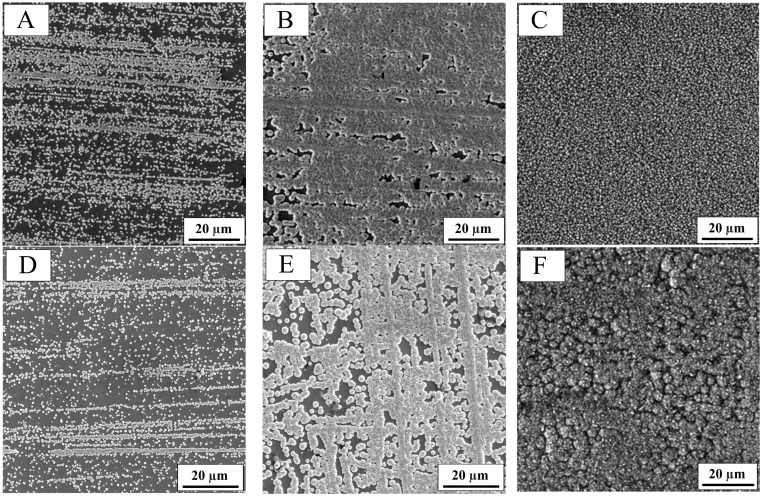
SEM images of alloy coating formation stages: (**A**) Co-Ni, thickness 0.1 µm, (**B**) Co-Ni, thickness 1 µm, (**C**) Co-Ni, thickness 5 µm, (**D**) Co-Ni(SBS), thickness 0.1 µm, (**E**) Co-Ni(SBS), thickness 1 µm, (**F**) Co-Ni(SBS), thickness 5 µm.

To observe crystal growth, successive layers with different theoretical thicknesses were deposited, followed by SEM imaging of each sample. The structures shown in Figure 2A–C represent co-deposition of cobalt and nickel, while the structures D–F correspond to alloy coatings deposited in the presence of SBS. Coatings A and D represent a thickness of 0.1 µm, B and E correspond to 1 µm, and C and F show coatings with a thickness of 5 µm. Analyzing the structure of the thinnest layer, spherical grains were observed in both cases, as well as the surface-blocking effect caused by SBS during deposition. A comparison of images B and E reveals variations in crystal growth between coatings deposited with and without the organic additive. The presence of SBS led to the formation of not only spherical grains but also elongated, sharp-ended structures. Further growth resulted in a uniform, fine-grained structure for the coating deposited without the additive (C), while the coating deposited with SBS (F) exhibited a more complex and varied surface morphology. 

SEM-EDS analysis was used to determine the elemental composition of the deposited coatings (5 µm), with the results summarized in Table 1. The presence of chromium and iron indicates a slight contribution of the substrate in the composition analysis, confirming the accuracy of the coating coverage. The sample deposited in the presence of SBS exhibits better substrate coverage, as the amounts of Cr and Fe are lower compared to the coating deposited without the additive.

Despite using a bath with a higher molar concentration of nickel, both coatings predominantly consist of cobalt (over 70%). A characteristic feature of nickel and cobalt co-deposition is the anomalous nature of this process. The obtained Co/Ni ratio confirms the preferential deposition of cobalt, even though it is a less noble metal [51]. The higher nickel content in the sample deposited without the additive is likely due to a greater contribution of the chromonickel substrate compared to the second sample.

**Figure 3:**
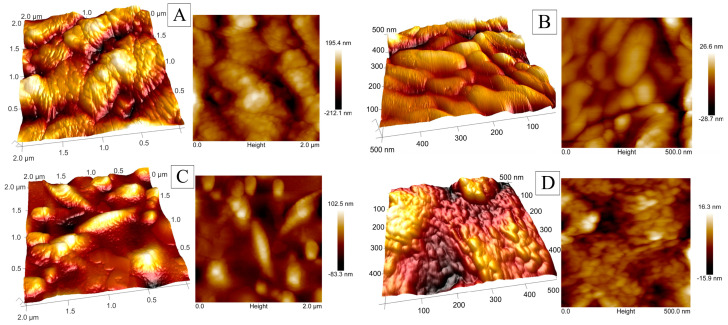
3D and 2D topography images of Co−Ni alloy coatings (**A**,**B**) and Co−Ni(SBS) organic additive deposited coatings (**C**,**D**) obtained by AFM at two magnifications.

To examine the surface topography and determine the roughness parameters of the obtained coatings, atomic force microscopy (AFM) was used. AFM images are presented in Figure 3 as three-dimensional (3D) and two-dimensional (2D) topography images. In both cases, the images confirm the presence of nanostructures with diameters below 100 nm. Similar to the SEM analysis, the same characteristic elongated, narrow islands were observed in the sample deposited with SBS. The analysis of surface profiles within an area of 500 × 500 nm (0.25 µm^2^) indicates that the organic additive influenced grain size, resulting in smaller grain diameters.

Surface roughness parameters were determined based on scans of areas measuring 0.25, 1, and 4 µm^2^. Table 2 presents numerical values for parameters such as Ra (average roughness), Rq (root mean square roughness), Rz (average distance between the highest peak and the lowest valley), Rp (maximum peak height), and Rv (maximum valley depth) [52]. The addition of SBS to the Co-Ni coating leads to a significant reduction in surface roughness. The decrease in roughness parameters in the Co-Ni(SBS) coating indicates a smoother structure with fewer surface irregularities. This effect may positively influence tribological properties and enhance resistance to mechanical wear. These findings confirm the effectiveness of SBS as an additive for reducing surface roughness, making it a valuable solution for applications requiring homogeneous and smooth surfaces. The hydrogen evolution process is complex, and its efficiency is influenced by many factors, not just structure. Smoother surfaces provide fewer active sites than more elaborate structures but reduce diffusion resistance, allowing more efficient release of hydrogen gas. On the other hand, rough surfaces can impede gas release due to increased diffusion resistance and surface interactions.

### 2.2. Determination and Comparison of Hydrogen Evolution Overpotential for Various Materials

The minimum potential required for hydrogen evolution in an alkaline environment, relative to the standard hydrogen electrode (SHE) at 25 °C, is −0.828 V at the cathode and 0.401 V at the anode, resulting in an approximate total of 1.23 V. The additional energy in the form of potential required to drive a given reaction at a specific rate is known as the overpotential [53]. The actual energy required to initiate the process under real conditions exceeds the theoretical minimum.

**Figure 4:**
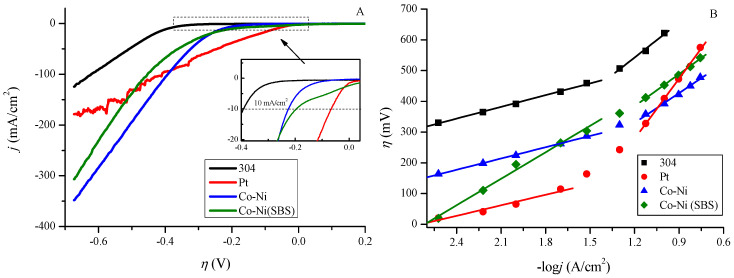
(**A**) Relationship between current density and overpotential for 304, Pt, Co−Ni, and Co−Ni(SBS) electrode obtained by linear voltammetry. (**B**) Dependence of overpotential as a function of the logarithm of current density. Parameters of fitted linear equations for low current densities (−log*j* from 2.5 to 1.5) and high current densities (−log*j* from 1.5 to 0.7) along with R^2^ coefficients are presented in Table 3.

To compare the overpotential of the deposited coatings with standard materials (304 stainless steel and Pt), current density was measured as a function of potential. The conversion of potentials relative to the reversible hydrogen electrode (RHE) allowed for the presentation of this relationship in terms of overpotential, as shown in Figure 4A. The recorded overpotential values at a current density of 10 mA/cm^2^ are 392, 225, 196, and 66 mV for 304 stainless steel, Co-Ni alloy coating, Co-Ni(SBS) coating, and platinum (Pt), respectively. The coating deposited with the SBS additive exhibits a lower overpotential in the low current density range. Based on Sabatier’s principle and the volcano plot [54], it is evident that platinum exhibits the best kinetic performance. However, as current density increases and new intermediate products form, active sites become blocked, leading to a decrease in HER efficiency. Therefore, the material must be sufficiently catalytic while also allowing for the facile desorption of reduction products [55]. Figure 4B presents the recorded overpotential values at specific current densities. The logarithmic representation of current density in this context is known as the Tafel relationship.

The dependence presented in Figure 4B reveals the presence of two distinct regions for all examined electrodes. In the low current density region, as expected, platinum exhibits the lowest overpotential, making it the most efficient material. As the current increases, the slope of the curve first rises for platinum at −log*j* = 2, followed by stainless steel 304 and Co-Ni at −log*j* = 1.4. A characteristic feature of the Co-Ni(SBS) coating is its ability to maintain a constant slope as the current density increases, which is a favorable operational property. In the high current density region, the slope increases significantly for the 304 and Pt electrodes (by 250.9 mV and 540.2 mV, respectively), while the increase is lower for the Co-Ni coating (201.0 mV) and the lowest for Co-Ni(SBS), at only 61.7 mV.

### 2.3. Hydrogen Evolution at Constant Voltage

**Figure 5:**
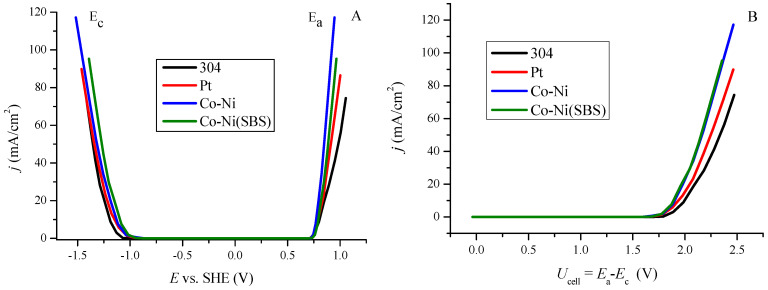
Dependence of current density as a function of individual potential values for cathode *E*_c_ and anode *E*_a_ (**A**) and hydrogen evolution process voltage (**B**).

Figure 5 presents the relationships determined by recording the current cathode potential (*E*_c_) and anode potential (*E*_a_) as a function of applied voltage (*U*) during electrolysis. Figure 5A represents the parameters for individual electrodes. When analyzing the performance of the cathode, the Co-Ni(SBS) coating exhibits the most favorable potential characteristics. On the other hand, in the case of the anode, stainless steel 304 demonstrates better performance, particularly when paired with the Co-Ni coating, which influences the overall electrode system performance. The curves in Figure 5B show the total work of the electrode array as a current density in the function of system voltage. Analysis of the curves indicates that the alloy coating enables a higher current density compared to stainless steel 304 and platinum at the same applied voltage. The current characteristics of the Co-Ni(SBS) coating are the same as those of the coating deposited without it, except for the maximum current density.

When using stainless steel 304 as the anode material, in addition to achieving higher current density in combination with the Co-Ni coating, a significant overpotential was observed on the anode side. The reactions occurring at the electrodes complement each other, meaning that overpotential at one electrode can limit the reaction at the other. This suggests that to improve system efficiency, the next step should involve modifying the anode, where the oxygen evolution reaction (OER) occurs in an alkaline environment.

### 2.4. Hydrogen Evolution at Constant Current

**Figure 6:**
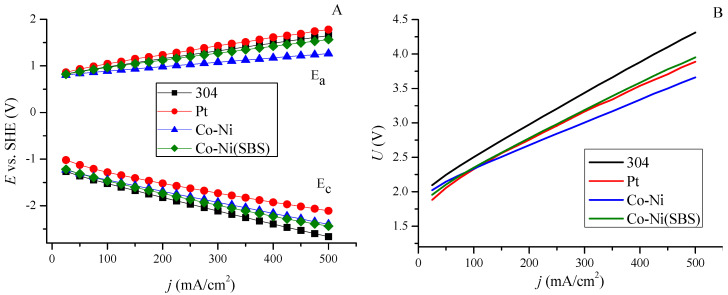
Dependence of electrode potential (**A**) and system voltage (**B**) as a function of current.

Hydrogen evolution was also studied under constant current conditions for all samples. The volume of evolved gases remained the same, as the supplied energy corresponded to the decomposition of a specific amount of water. Measurements were conducted by separately recording the cathode and anode potentials. This allowed for an evaluation of the cathode–anode system performance, highlighting differences in potential requirements to sustain a given current, as shown in Figure 6A. Platinum, as a cathode, exhibits the most favorable properties, whereas anode performance is limited and insufficient. The cathode–anode system operates most efficiently with the Co-Ni coating. The overall system performance is presented in Figure 6B. In the initial stage, at a current density of 25 mA/cm^2^, platinum requires the lowest energy input in the form of voltage. However, this trend changes above 100 mA/cm^2^, where the alloy coating deposited without additives requires less energy input. The 304 stainless steel sample exhibits the highest voltage values across the entire investigated current range.

### 2.5. Efficiency of Structures in the Hydrogen Evolution Process

**Figure 7:**
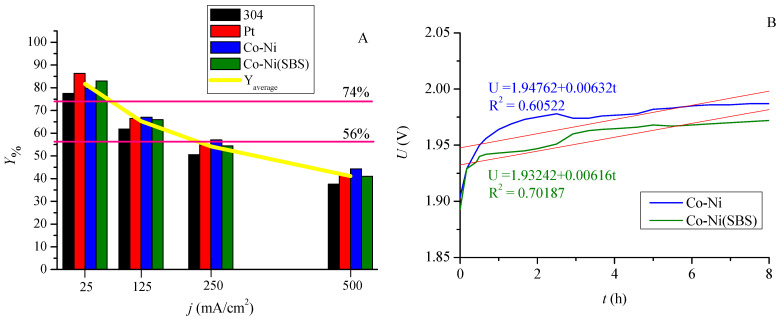
(**A**) Hydrogen evolution yields of individual materials measured at current densities of 25, 125, 250, and 500 mA/cm^2^. (**B**) Stability of alloy coatings at 10 mA/cm^2^ during an eight-hour hydrogen evolution process.

The previously described characteristics translate into material efficiency, as illustrated in Figure 7A. Under low-current and low-potential conditions, platinum exhibits the highest efficiency. However, hydrogen evolution under these conditions is slow and economically unfeasible. In industrial applications, the focus is on designing electrolyzers that operate at high current densities. As shown in the results, the efficiency of platinum decreases significantly with increasing current density. Therefore, Co-Ni alloy coatings are a more cost-effective solution, both in terms of material costs and performance improvements. An eight-hour hydrogen evolution process at a current density of 10 mA/cm^2^ was conducted by recording voltage variations over time, as presented in Figure 7B. The regression line-derived R^2^ parameter indicates better stability for the coating deposited with the additive. Based on this observation, it can be concluded that the presence of the additive during deposition allows the maintenance of the properties of Co-Ni alloy coatings in the electrolysis environment.

The measurement of the gas volume generated during electrolysis provided a set of performance parameters for the coatings, offering a quantitative assessment of material quality and enabling comparisons with other electrolyzer configurations. The individual values are listed in Table 4. These parameters represent the energy efficiency of the process, calculated using different units. Current technological solutions achieve efficiencies in the range of 56–74%. Depending on the applied current density and material, the constructed system achieves comparable values. Operating at a current density of 500 mA/cm^2^, the system reaches an efficiency of approximately 40%. It is important to note that the results were obtained under laboratory conditions at room temperature. The developed alloy coatings could be implemented in industrial electrolyzers as alternative materials to stainless steel plates and platinum-based surfaces.

## 3. Materials and Methods

### 3.1. Preparation of the Substrate for Deposition

A chromonickel stainless steel sheet (304, Eurostal-Metale S.A., Rzeszow, Poland) was electropolished (15 min) in a solution containing 300 cm^3^ of 85% H_3_PO_4_ (p.a., CHEMPUR, Piekary Śląskie, Poland), 185 cm^3^ of concentrated H_2_SO_4_ (p.a., CHEMPUR), and 15 cm^3^ of distilled water, using a current density of 21 A/dm^2^.

### 3.2. Electrodeposition

The electropolished sheet was degreased with acetone and placed in a thermostated electrolyzer (25 °C) containing an electrolyte bath suitable for the deposited material. The qualitative and quantitative compositions of the electrolytes are presented in Table 5, where the first row corresponds to the coating deposited without additives, referred to hereafter as Co-Ni. The second row represents the electrolyte composition for the coating deposited with the SBS additive, designated as Co-Ni(SBS). All compounds were of analytical grade; metal salts were sourced from CHEMPUR, while SBS was obtained from TCI.

The samples were deposited using PGSTAT100 (Autolab) and Epsilon (BASi) apparatus with the chronopotentiometric (CP) technique, applying a current density j of 1 A/dm^2^. The chromonickel (304) substrate served as the working electrode, nickel plates were used as counter electrodes, and a saturated calomel electrode (SCE) was employed as the reference electrode. The theoretical coating thickness was 5 µm. After metal deposition, the coatings were rinsed with distilled water and dried.

The coatings were analyzed using SEM-EDS (Hitachi S-3400, Hitachi High-Technologies Corporation, Tokyo, Japan) to determine their composition. Topography and surface roughness parameters were studied using a Nanoscope V atomic force microscope (Bruker) with NanoScope 5 Analysis software. Topography mapping was performed using the TappingMode technique, using an RTESPA-300 needle, with a resonance frequency of 300 kHz, a scanning speed of 0.5 Hz, and a resolution of 512 lines.

### 3.3. Electrolytic Hydrogen Evolution

To determine the hydrogen evolution overpotentials, an Epsilon (BASi) apparatus was used. The polarization curves were recorded at a scan rate of 50 mV/s. A 1 M NaOH solution (p.a., CHEMPUR) was used as the electrolyte.

A laboratory setup was designed and constructed to conduct electrolysis while simultaneously collecting the gases evolved at the cathode (H_2_) and anode (O_2_). The same electrolyte was used as mentioned above. A chromonickel plate served as the anode. The current *i* and the potentials of the cathode *E*_c_ and anode *E*_a_ were recorded while varying the voltage *U*. Subsequently, at a constant voltage over a specified time, the volume of the evolved gases was measured. In the second technique, *E*_c_ and *E*_a_ were measured at fixed current values.

## 4. Conclusions

1. The electrodeposition of alloy coatings enabled the development of materials functioning as cathodes in the electrolysis process. The influence of sodium benzenesulfonate as an additive in the co-deposition process was also examined. The coating structures exhibited complete surface coverage, while the additive facilitated the formation of grains with varying shapes and sizes. The obtained results confirm the effectiveness of SBS in reducing surface roughness.

2. The hydrogen evolution overpotential values were lower than those of stainless steel but higher compared to platinum. The addition of SBS contributed to a reduction in the overpotential of the Co-Ni cathode.

3. Depending on the applied current density, it is possible to determine the material that ensures the highest efficiency of electrolytic hydrogen evolution. Under higher current densities typical for industrial electrolyzers, the alloy coatings demonstrated the best performance.

4. The application of sodium benzenesulfonate enhances the stability of the catalytic material during hydrogen evolution.

## Figures and Tables

**Table 1:** Elemental percentage composition [% wt] and standard deviation (SD) of samples based on SEM-EDS analysis.

Coating Designation	Elemental Content [% wt] ± SD						Co/Ni
	C	O	Cr	Fe	Co	Ni	
Co-Ni	2.06 ± 0.93	1.30 ± 0.74	0.50 ± 0.04	0.61 ± 0.08	70.23 ± 0.91	25.30 ± 0.44	2.78
Co-Ni(SBS)	1.51 ± 0.49	0.80 ± 0.38	0.22 ± 0.03	0.25 ± 0.03	72.65 ± 0.52	24.58 ± 0.57	2.95

**Table 2:** Individual surface roughness parameters of coatings obtained by AFM.

Roughness Parameter	Coating Designation	Scanning Surface [µm^2^]		
		0.25	1	4
R_a_ [nm]	Co-Ni	5.61	16.0	47.5
	Co-Ni(SBS)	2.90	8.17	18.3
R_q_ [nm]	Co-Ni	7.17	20.7	58.4
	Co-Ni(SBS)	3.71	10.8	24.7
R_z_ [nm]	Co-Ni	8.07	52.9	122
	Co-Ni(SBS)	7.18	28.2	67.6
R_p_ [nm]	Co-Ni	25.1	62.4	179
	Co-Ni(SBS)	14.4	42.8	121
R_v_ [nm]	Co-Ni	−34.9	−87.9	−198
	Co-Ni(SBS)	−16.0	−41.7	−83.0

**Table 3:** Determined Tafel parameters.

Sample Determination	Low Current Density Values			High Current Density Values		
	Tafel Slope [mV]	−log*j*_0_ [A/cm^2^]	R^2^	Tafel Slope [mV]	−log*j*_0_ [A/cm^2^]	R^2^
304	127.3	5.5	0.9985	378.2	8.4	0.9947
Pt	114.2	2.6	0.9793	654.4	9.0	0.9979
Co-Ni	122.4	4.0	0.9992	323.4	6.1	0.9965
Co-Ni(SBS)	287.1	6.4	0.9955	348.8	6.8	0.9987

**Table 4:** Parameter of hydrogen evolution efficiency of individual surfaces, measured at current density 25, 125, and 250 mA/cm^2^.

Determination of the Cathode Area	Current Density [mA/cm^2^]	Hydrogen Evolution Yield Parameter				
		gH_2_/kWh	kWh/kgH_2_	kJ/molH_2_	kWh/molH_2_	Y_%_
304	25	19.68	50.8	369.2	0.103	77.50
	125	15.70	63.7	462.8	0.129	61.83
	250	12.85	77.8	565.6	0.157	50.59
	500	9.55	104.7	760.9	0.212	37.60
Pt	25	21.91	45.6	331.7	0.092	86.27
	125	16.89	59.2	430.3	0.120	66.49
	250	13.92	71.8	521.9	0.145	54.83
	500	10.59	94.4	686.0	0.191	41.71
Co-Ni	25	20.26	49.4	358.6	0.100	79.79
	125	17.01	58.8	427.2	0.119	66.99
	250	14.49	69.0	501.6	0.139	57.05
	500	11.25	88.9	645.9	0.180	44.30
Co-Ni(SBS)	25	21.08	47.4	344.7	0.096	83.01
	125	16.74	59.7	434.0	0.121	65.93
	250	13.81	72.4	526.1	0.146	54.39
	500	10.42	96.0	697.2	0.194	41.04

**Table 5:** Qualitative and quantitative compositions of electrolytes used.

Coating Designation	$c_{{NiCl}_{2}}$ [mol/dm^3^]	$c_{{CoCl}_{2}}$ [mol/dm^3^]	$c_{SBS}$ [%]	$c_{{H_{3}BO}_{3}}$ [mol/dm^3^]
Co-Ni	0.06	0.04	-	0.5
Co-Ni(SBS)			1	

## Data Availability

The original contributions presented in this study are included in the article. Further inquiries can be directed to the corresponding authors.

## Abbreviations

The following abbreviations are used in this manuscript:

AFM	Atomic force microscopy
CP	Reversible hydrogen electrode
HER	Hydrogen evolution reaction
OER	Oxygen evolution reaction
RES	Renewable energy sources
RHE	Reversible hydrogen electrode
SBS	Sodium benzene sulfonate
SCE	Saturated calomel electrode
SEM	Scanning electron microscope
SHE	Standard hydrogen electrode
